# Vaginal Microbiome and Its Relationship with Assisted Reproduction: A Systematic Review and Meta-Analysis

**DOI:** 10.3390/life15091382

**Published:** 2025-09-01

**Authors:** Marise Samama, Joji Ueno, Eduardo Carvalho de Arruda Veiga, Rita C. C. P. Piscopo, Fabio Ikeda, Nina Pires de Lemos, Lucas Tadeu Bidinotto, Márcia Guimarães da Silva, Zsuzsanna Jarmy Di Bella, Frida Entezami

**Affiliations:** 1 Department of Gynecoloy, Escola Paulista de Medicina, Federal University of São Paulo-UNIFESP, São Paulo 04024-002, Brazil; marisesamama@yahoo.com.br (M.S.); zsuvi@uol.com.br (Z.J.D.B.); 2 IVF Department, GERA Institute for Teaching and Research Reproductive Medicine São Paulo, São Paulo 01409-001, Brazil; jojiueno@uol.com.br (J.U.); eduardo.veiga@fm.usp.br (E.C.d.A.V.); draritapiscopo@gmail.com (R.C.C.P.P.); famikeda@uol.com.br (F.I.); ninalemos29@gmail.com (N.P.d.L.); 3 Department of Obstetrics and Gynecology, Discipline of Gynecology, Hospital das Clínicas, Faculty of Medicine-University of São Paulo, São Paulo 05403-000, Brazil; 4 Barretos School of Health Sciences Dr. Paulo Prata-FACISB, Barretos 14785-002, Brazil; lucasbidinotto@gmail.com; 5 Molecular Oncology Research Center, Barretos Cancer Hospital, Barretos 14784-400, Brazil; 6 Department of Pathology, Botucatu Medical School, São Paulo State University-Unesp, Botucatu 18607-340, Brazil; marcia.guimaraes@unesp.br; 7 IVF Department, American Hospital of Paris, 92200 Neuilly-sur-Seine, France; 8 UFR Simone Veil Santé, University UVSQ, Paris-Saclay, 78180 Montigny-le-Bretonneux, France

**Keywords:** vaginal microbiome, clinical pregnancy rate, live birth rate, pregnancy loss, systematic review

## Abstract

**Background**: The vaginal microbiome is an important factor influencing clinical outcomes in women undergoing assisted reproductive techniques. **Objective**: Our review aimed to confirm that women with a favorable vaginal microbiome have better reproductive outcomes than women who have an unfavorable vaginal microbiome. **Methods**: This systematic review and meta-analysis included articles published in the last 10 years, identified through keyword searches in PubMed and MEDLINE using the MeSH terms “vaginal microbiome,” “reproduction,” and “human reproduction.” The search yielded 1735 records. Participants were categorized into women with a favorable microbiome and those with an unfavorable microbiome. To refine the taxonomic resolution at the species level, we additionally performed a bioinformatic analysis of a cohort of 56 women using multivariable association with linear models (MaAsLin2). **Results**: Women with a favorable microbiome had higher pregnancy rates compared to those with a less favorable microbiome (*p* = 0.0001, I2 = 0%, RR: 1.59). Live birth rates were also significantly higher in the favorable microbiome group than in the unfavorable microbiome group (*p* = 0.004, I2 = 0%, RR:1.41), with no evidence of heterogeneity. Women with an unfavorable microbiome had more miscarriages than women with a favorable microbiome (*p* = 0.04, I2 = 0%, RR: 0.65). Bioinformatic analysis showed that a high relative abundance of *Lactobacillus crispatus* increased the likelihood of pregnancy approximately sixfold. **Conclusions**: The favorable microbiome group, particularly participants with a high relative abundance of *Lactobacillus crispatus*, demonstrated better reproductive outcomes, with a higher clinical pregnancy rate, a higher live birth rate, and a lower rate of pregnancy loss, although there was a low-quality bias.

## 1. Introduction

Infertility treatment results in thousands of in vitro fertilization (IVF) cycles annually. A global analysis showed an average delivery rate from assisted reproductive technology (ART) treatments of 19% per aspiration [1]. Most unsuccessful ART treatments remain unexplained. A comprehensive assessment of infertility etiologies could bring better understanding and higher live birth rates (LBRs) in ART. Analysis of the genital microbiota has emerged as an important tool for the diagnosis of infertility. Initially described via microscopy, which can be influenced by the examiner’s subjective assessment, the microbiota was later characterized using molecular methods such as quantitative polymerase chain reaction (qPCR), targeting selected bacteria related to infertility [2], and more recently with next-generation sequencing (NGS).

The study of the composition of the vaginal microbiome and its molecular characterization has been increasingly refined over the past 15 years. The vaginal microbiome has been associated with sexually transmitted infections (STIs), cervical dysplasia, and, more recently, fertility and pregnancy outcomes like miscarriage and preterm birth. A disrupted vaginal microbiome appears to be one of the causes of ART failure.

The vagina is predominantly inhabited by *Lactobacillus*, which help prevent infections and constitute the major component of the vaginal microbiome. The composition and structure of the microbiome, analyzed using molecular techniques such as next-generation sequencing (NGS) of the 16S rRNA gene,, have been classified into five bacterial community state types (CSTs) of taxonomic composition, reflecting patterns in the relative abundance of different genomic species and their associated environmental conditions [3]. Four CSTs are dominated by species of the *Lactobacillus* genus: *L. crispatus* (CST I), *L. gasseri* (CST II), *L. iners* (CST III), and *L. jensenii* (CST V), whereas CST IV is characterized by a paucity of *Lactobacillus* spp. and a diverse array of strict and facultative anaerobes. CST IV comprises most cases of bacterial vaginosis and is a major risk factor for the acquisition and transmission of STIs. The composition of the vaginal microbiome can vary across different ethnicities and geographic locations. Moreover, behavioral and lifestyle factors, including intimate hygiene practices, alcohol consumption, and smoking, have been independently associated with a *Lactobacillus*-depleted vaginal microbiota, which may be useful indicators of women at increased risk of having a disrupted vaginal microbiome [4]. The vaginal microbiome also changes with age and menstrual cycle phase, with *Lactobacillus* abundance increasing during the proliferative phase due to high estrogen levels. In addition to these factors, STIs and vaginal pathogens can further disrupt the vaginal microbiome, increasing the risk of reproductive tract infection [4]. A dysbiotic vaginal microbiome may damage the epithelial cervical barrier, promote endometrial infection, and affect early pregnancy [5]. The uterine microbiome, in contrast to the vaginal microbiome, has 10000 times fewer bacteria, and there is a debate on whether a core microbiome really exists due to the risk of contamination when accessing the endometrial cavity. Cicinelli et al. (2015) [6] demonstrated that chronic endometritis was associated with high bacterial diversity and recurrent implantation failure. Some studies have demonstrated that, in chronic endometritis, lactobacilli are less abundant, and there is an increase in the prevalence of genera such as Bacteroides, *Streptococcus*, *Staphylococcus*, *Gardnerella*, *Neisseria*, and *Klebsiella* [7]. Chronic endometritis and endometriosis have been described as diseases that cause infertility and involve inflammation and immune dysregulation. Some studies suggest that a disrupted microbiome may influence the development of both pelvic inflammatory disease and endometriosis [8]. Polycystic ovary syndrome is a complex and multigenic metabolic disorder that causes anovulation affecting the menstrual cycle and fertility, while some data also suggest a role for altered gut microbiome and a decreased relative abundance of *Lactobacillus* in the vaginal microbiome. Lee et al. showed significant associations between microbiomes from the reproductive tract with fasting insulin, and there were significant changes in microbial diversity across the menstrual cycle [9].

In the context of reproductive health, the vaginal microbiome may be characterized as healthy or unhealthy, based on its taxonomic composition, relative abundance, environment, and how it affects assisted reproduction and pregnancy [10,11].

A healthy vaginal microbiota maintains eubiosis and an acidic environment with a pH ranging between 3.5 and 4.5 [11,12] that is protective against STIs and is favorable to fertility and pregnancy outcomes. *Lactobacillus* species are among the most common bacteria in the vagina and are widely considered hallmarks of vaginal health. In women with vaginal dysbiosis and increased diversity, the loss of *Lactobacillus* dominance and lack of lactic acid promotes a higher pH. A disrupted vaginal microbiome can be composed of an unfavorable bacterial environment formed by species such as *Gardnerella vaginalis*, *Fannyhessea vaginae*, *Prevotella* spp., and *Sneathia* spp. [12,13]. These pathogens are associated with bacterial vaginosis (BV), the most prevalent vaginal dysbiosis, which is characterized by a decrease in *Lactobacillus* and an increase in anaerobic bacteria and has been associated with recurrent implantation failure. According to the taxonomic classification, CST I, II, III, and V are classified as favorable female microbiomes in human reproduction and CST IV as unfavorable [14,15].

Furthermore, the estrogen-dominant vaginal epithelium is essential for the survival and growth of *Lactobacillus* spp. These produce anti-inflammatory cytokines and antimicrobial peptides from epithelial cells, strengthening the epithelial cell barrier which contributes to genital health [16] by reducing oxidative stress and regulating vaginal microbiota composition. It is hypothesized that the hormones used during assisted reproduction could influence the vaginal microbiome through glycogen accumulation, which is utilized by *Lactobacillus*. Interestingly, Zhao C et al. (2020) [17] demonstrated that the vaginal microbiome in infertile women undergoing in vitro fertilization had a significant decrease in microbiome diversity and richness. They showed an increased abundance of *Atopobium*, *Aerococcus*, and *Bifidobacterium* and decreased abundance of *Lactobacillus*, and no changes were observed during the follicular phase under conditions of gonadotropin-releasing hormone (GnRH) agonist and recombinant human chorionic gonadotropin (r-hCG) induction. They concluded that the vaginal microbiome of infertile women is not sensitive to hormonal changes during controlled ovarian stimulation (COS). On the other hand, Carosso et al. [18], analyzing the vaginal and endometrial microbiome after COS during the luteal phase, supported with progesterone (P) at the embryo transfer, reported a small, but not significant, decrease in *Lactobacilli* and higher bacterial diversity. In the vagina, they observed an increase in pathogenic species including *Prevotella* and *Escherichia coli–Shigella* spp. In the endometrium, the proportion of *Lactobacilli* slightly decreased, while both *Prevotella* and *Atopobium* increased. They suggest that COS and P supplementation change the composition of the vaginal and endometrial microbiota, concluding that this instability could affect both endometrial receptivity and placentation. In 2024, a study [19] monitoring the vaginal microbiome during multiple consecutive fertility treatments (pretreatment and around ovulation) in a subfertile population undergoing IUI and IVF identified a decrease in *Lactobacilli*, potentially leading to a higher incidence of bacterial vaginosis. This decrease in *Lactobacilli* and the negative change in microbiome status could reduce ongoing pregnancy rates. A particular strength of this study was the multiple observations made during consecutive treatment cycles.

During pregnancy, there is a decline in the metabolic capacity of the vaginal resident microbiome, which is consistent with a change to a less complex *Lactobacillus*-dominated microbiome [20], decreasing in diversity. Hormonal changes are responsible for these changes in the microbiome, especially due to a higher rise in progesterone levels. During early successful pregnancy, *Lactobacillus iners* is the most detected bacterium and plays an important role in defense. *Lactobacillus crispatus* maintains a healthy environment during pregnancy. Some studies suggest that most miscarriages belong to the CST not dominated by *Lactobacillus* and with imbalanced cytokines that may promote pregnancy loss [20]. After mid-pregnancy, disruption of the vaginal microbiota is the most significant risk factor for preterm delivery before 37 weeks of gestation. A recent study demonstrated that the relative abundance of beneficial bacteria, such as *Lactobacillus*, was significantly reduced in pregnant women who experienced preterm birth compared to a group who delivered at term [21]. Currently, physical and biochemical markers are commonly used to evaluate preterm pregnancy outcomes but have limited accuracy.

A successful pregnancy depends on the crucial embryo–maternal interaction and the reasons for most cases of unsuccessful ART remain unexplained. As the vaginal microbiome seems to play an important role in human reproduction, the objective of this study was to observe the relationship between ART and the vaginal microbiome in real life, by developing a systematic review and meta-analysis. For the purposes of this systematic review, the authors classified the favorable microbiome according to the taxonomic classification cited above as CST I, II, III, and V and CST IV as the unfavorable microbiome.

## 2. Materials and Methods

The PRISMA guidelines were followed in this review, and the search strategy followed the article by Page et al. [22]. The PubMed, Medline, and SciELO databases were searched using the MeSH terms “vaginal microbiome”, “reproduction”, and “human reproduction”. The inclusion criteria were articles in English published in the period from July 2014 to December 2024. The exclusion criteria were animal studies, articles without vaginal microbiome in the title, articles without vaginal microbiome in the abstract, and review articles or systematic reviews. This resulted in 1741 articles being identified in the first round. The titles and summaries of the articles were then read, and those not meeting the inclusion criteria were excluded (animal studies, *n* = 170; articles without vaginal microbiome in the title, *n* = 678; articles without vaginal microbiome in the abstract, *n* = 678; and review articles or systematic reviews, *n* = 196). Any duplicate articles were then excluded (*n* = 6). At the end of this process, there were 19 articles left for full-text reading. Of these, 11 articles were excluded as they did not meet the objective of this systematic review, and 8 studies were chosen for the systematic review and meta-analysis.

**Figure 1:**
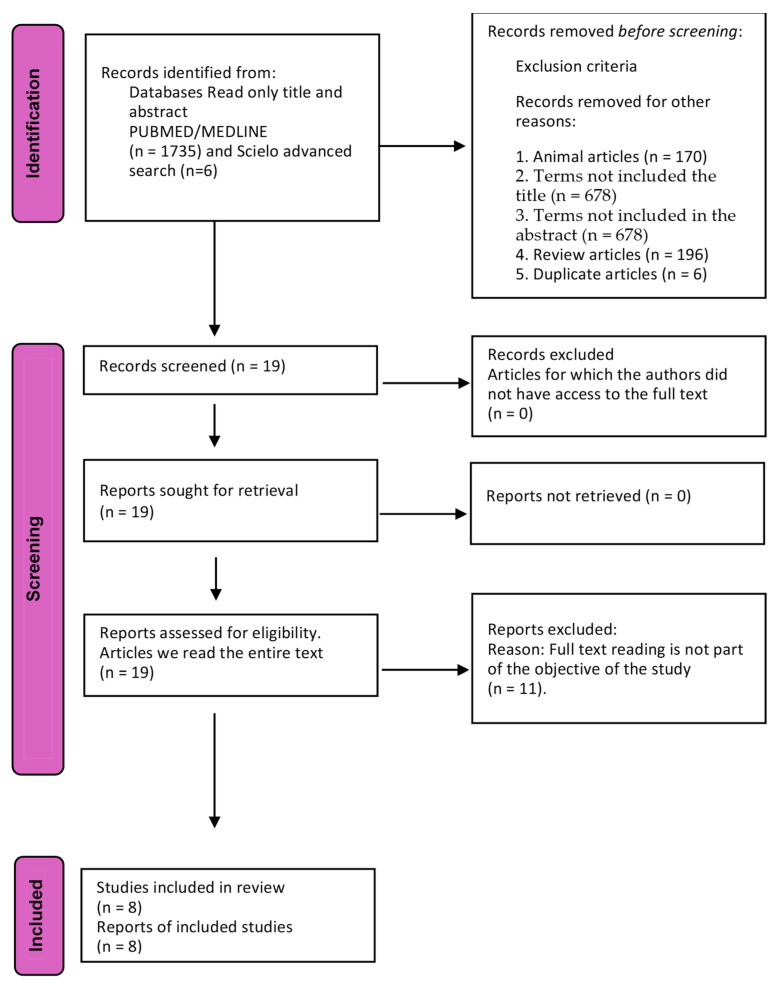
Flowchart of selected studies. From [22].

Two different researchers independently conducted the search strategy (MS and ECAV) using the POS framework: P for patients, O for outcomes, and S for study design (POS). Women were classified as either having a favorable or unfavorable microbiome according to the CST classification, and the outcomes considered were clinical pregnancy rates (CPRs) and live birth rates (LBRs). S was the type of study, and none of the 8 selected studies were cohort development, cohort prospective, non-randomized clinical trial, or observational studies (Figure 1) [22,23].

Determination of the species associated with a favorable outcome:

In order to determine which species were associated with a successful pregnancy, we intended to download sequencing raw data from the eight studies and re-analyze them, using “successful pregnancy” as an endpoint and applying a regression analysis to determine those taxa which were statistically associated with this endpoint. However, of the eight studies, only Väinämö et al. (2023) [24] had raw data and metadata deposited in the Sequence Read Archive (SRA) repository (https://www.ncbi.nlm.nih.gov/sra/PRJEB61794, accessed on 8 April 2025).

This, to avoid any methodological biases, we obtained sequencing data only from the MiSeq42 library, from material obtained only at the time of fresh IVF-ET (*n* = 76). Next, we ran dada2 RevMan Web [25] using default parameters and eliminated those sequencing which had low count numbers. We then analyzed the sequencing of 56 patients, 23 with successful pregnancy and 33 with unsuccessful pregnancy. Finally, we applied Microbiome Multivariable Association with Linear Models (MaAsLin2) [26] for determining the relevant taxa associated with successful pregnancy.

## 3. Results

**Study Selection and Participant Characteristics**

After analyzing the full text of the 19 selected articles, 11 articles did not fit the objectives of our study, therefore, 8 articles were selected to enter the systematic review and meta-analysis (Figure 1) [24,25,26,27,28,29,30,31,32,33]. The total number of women evaluated in these meta-analyses was 942 participants.

Table 1 shows the main characteristics of the eight studies. The average age of the participants was 33.53 years, indicating that the studies focused on women who were at a reproductive age appropriate for IVF. The average body mass index (BMI) value was 25.59, which indicates that the study participants were of normal weight for assisted reproduction. The average individual anti-Mullerian hormone (AMH) level was 2.76 (ng/mL), which is considered normal for ovarian reserve.

**Vaginal Microbiome Composition and Reproductive Outcomes**

The relationship between *Lactobacillus* spp. abundance and reproductive outcomes varied across studies. Eskew et al. (2021) [27] reported that the bacterial community was not a reliable predictor of the timing of embryo transfer, underscoring the need to assess the vaginal microbiome at the appropriate time point. In contrast, several studies observed that higher *Lactobacillus* abundance was associated with favorable outcomes: Karaer et al. (2021) [28] and Koedooder et al. (2019) [29] found that women with pregnancy failure had lower *Lactobacillus* levels than those with successful pregnancies; Miyagi et al. (2023) [30] reported a higher abundance in pregnant women; and Vainamo et al. (2023) [24] showed that *L. crispatus* was linked with greater IVF success. Consistent with this, Vergano et al. (2019) [31] reported that higher *L. crispatus* abundance was associated with live birth rate. By contrast, Koort et al. (2023) [32] found that *L. iners* and *L. gasseri* communities were less successful in assisted reproduction compared with *L. crispatus*. Similarly, van den Tweel et al. (2024) [33] reported a greater tendency for abortion in CST III and IV communities, which are characterized by low *Lactobacillus* dominance.

**Causes of Infertility in the Included Studies**

The causes of infertility varied across studies (Table 1). Male factor was the most frequently reported, followed by tubal factor, endometriosis, and unexplained infertility. Some studies reported multiple causes (e.g., Vainamo et al., 2023 [24]), while others provided no details (e.g., Koort et al., 2023 [32]; Vergano et al., 2019 [31]).

**Meta-analysis of Live Birth and Pregnancy Outcomes**

**Figure 2:**
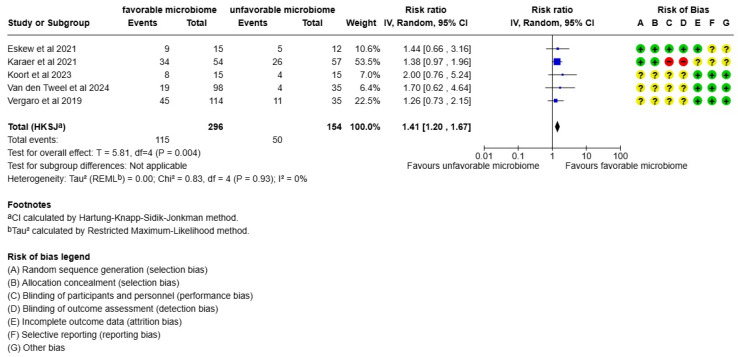
Meta-analysis of the live birth rate of women with a favorable microbiome compared to women with an unfavorable microbiome. The references in Figure 2 are Eskew et al., 2021 [27], Karaer et al., 2021 [28], Koort et al., 2023 [32], van den Tweel et al., 2024 [33], and Vergaro et al., 2019 [31].

The main outcome of the result of the meta-analysis when evaluating live birth rates was that women who were in the most favorable microbiome group had higher and statistically significant chances of a successful pregnancy (*p* = 0.004, I2 = 0%, RR: 1.41) (Figure 2), without any heterogeneity.

**Figure 3:**
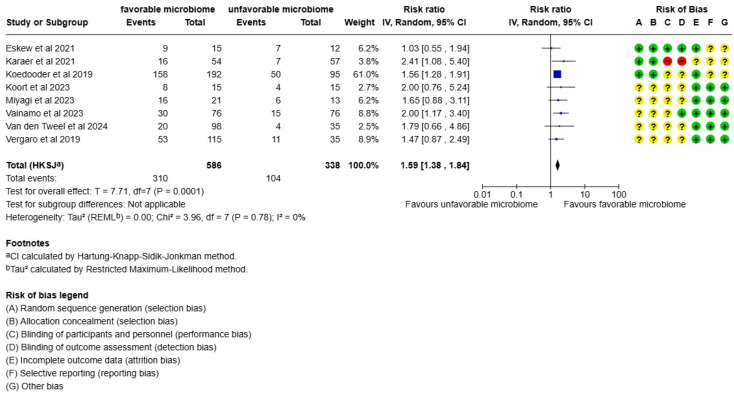
Meta-analysis of the clinical pregnancy rate of women with a favorable microbiome compared to women with an unfavorable microbiome. The references in Figure 3 are Eskew et al., 2021 [27], Karaer et al., 2021 [28], Koedooder et al., 2019 [29], Koort et al., 2023 [32], Miyagi et al., 2023 [30], Vainamo et al., 2023 [24] van den Tweel et al., 2024 [33], and Vergaro et al., 2019 [31].

Focusing on clinical pregnancy rate results, women in the more favorable vaginal microbiome group also experienced higher pregnancy rates (*p* = 0.0001, I2 = 0%, RR:1.59) compared to the women with a less favorable microbiome (Figure 3).

**Figure 4:**
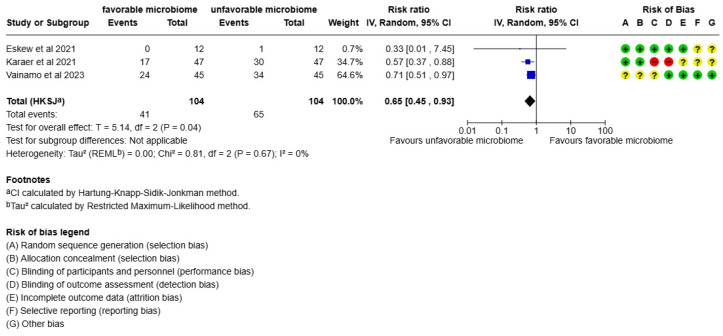
Meta-analysis comparing pregnancy loss between women with favorable microbiome and women with unfavorable microbiome. The references in Figure 2 are Eskew et al., 2021 [27], Karaer et al., 2021 [28], and Vainamo et al., 2023 [24].

When pregnancy losses were analyzed, statistics showed that women with an unfavorable microbiome had more miscarriages than women with a favorable microbiome (*p* = 0.04, I2 = 0%, RR: 0.65) (Figure 4).

**Risk of Bias Assessment**

Risk of bias was assessed for the eight studies included in this review using the Cochrane tool for non-randomized clinical trials. Seven domains were evaluated. For *random sequence generation*, 37.5% of studies were judged to have a low risk of bias and 62.5% an unclear risk. For *allocation concealment*, 37.5% were considered low risk and 62.5% unclear. For *blinding of participants and personnel* as well as *blinding of outcome assessment*, 12.5% were rated high risk, 12.5% low risk, and 75% unclear. For *incomplete outcome data*, 87.5% were low risk and 12.5% unclear. Finally, for *selective reporting* and *other sources of bias*, 62.5% were low risk and 37.5% unclear.”

When all seven domains across the eight studies were considered together, 46.4% of judgments indicated a low risk of bias, while 50% were rated as unclear. For *allocation concealment*, most studies (70%) were judged to have an unclear risk of bias, with only a small proportion (3.6%) rated as low risk. Overall, these results suggest that many of the included studies had important methodological limitations, particularly related to unclear or high risks of bias, which must be taken into account when interpreting the findings.

**Additional Analysis of Väinämö et al. (2023)**

A detailed reanalysis of Väinämö et al. (2023) [24] found 35 taxa represented in the cohort at the genus and/or species level. MaAsLin2 analysis showed that, from all these taxa, the high relative abundance of *Lactobacillus crispatus* was associated with a successful pregnancy (*p* = 0.003) with a coefficient = 1.802, which represents about a 6 times greater chance of a successful pregnancy (Table 2).

## 4. Discussion

The main findings of the study indicate that women with a favorable microbiome had higher pregnancy and live birth rates, although the included studies were subject to a high risk of bias. Celicanin et al. [34] in a review of twenty-five studies on women with vaginal dysbiosis (mostly analyzed by microscopy with only three studies using molecular analysis) reported lower clinical pregnancy rates and increased pregnancy loss rate. Another important result was that women who had an unfavorable microbiome, according to the CST classification, had fewer pregnancies (RR: 1.59), a result that is supported by our review. We also observed significantly lower live birth rates in women who had vaginal dysbiosis (RR: 1.41), a conclusion which the authors of the previous review could not draw due to the high heterogeneity between the studies. We also observed a higher miscarriage rate in participants with an unfavorable microbiome (RR: 0.65). 

Similar trends were observed in the meta-analysis and systematic review by Skafte-Holm et al. [35] in women with vaginal dysbiosis, mostly analyzed by microscopy, which reported low pregnancy rates, although there was no significant difference in live birth rates, suggesting that molecular methods provide more detailed insights into the vaginal microbiota [36]. In 2019, Haar et al. [37], in another meta-analysis of studies using mostly microscopy, found no significant association between bacterial vaginosis and clinical pregnancy rates and live birth rates. However, the authors attributed a high risk of bias to most of the studies. In another systematic review, Gudnadottir et al. [36] found that women with a lack of *Lactobacillus* spp. dominance, classified as an unfavorable microbiome, had a higher risk of premature birth (OD: 1.69).

Some studies have shown that the vaginal microbiome associated with eubiosis and the dominance of *Lactobacillus* spp. presents better chances of embryo implantation and pregnancy in ART. Vaginal dysbiosis can cause major local inflammation by increasing pro-inflammatory cytokines, which compromise endometrial receptivity and induce recurrent implantation failure and pregnancy loss [4,38,39]. Other studies focused on obstetric results, such as pre-eclampsia [40], and premature birth, demonstrating that women with an unfavorable microbiome are more likely to have preterm births compared to women with a favorable microbiome. A 2023 meta-analysis [41] concluded that women with reduced vaginal *Lactobacillus* spp. have an increased risk (OR: 1.69) of preterm birth and another study has found that the vaginal microbiome was a better predictor of preterm labor than premature birth [16]. Recent studies suggest that a dysbiotic microbiome combined with altered estrogen metabolism can contribute to the immunopathogenesis of endometriosis, which is strongly related to infertility [42].

Results on the relative abundance of *Lactobacillus* in the studies selected for this systematic review showed that better reproductive outcomes were associated with a greater abundance of *Lactobacillus* spp. in pregnant women. Specifically, *L. crispatus* was the species that presented the greatest abundance in women who underwent in vitro fertilization with successful pregnancy, and this agrees with recent literature. Wright et al. (2021) [43] found that a prevalence of *L. crispatus* is dominant among women of European ancestry but not among women of African ancestry. A systematic review and analysis by Incognito et al. (2025) [44] explored the relationship between cervicovaginal microbiota diversity and *Lactobacillus* profiles and their relationship with HPV and invasive cervical cancer. The authors, after performing the meta-analysis and analyzing the results, demonstrated that the relationship of *Lactobacillus* species, particularly *L. crispatus*, could be seen as a biomarker of health for the cervicovaginal microbiota but also as a biomarker for the risk of cancer progression and suggested that further studies in this area need to be carried out to validate the use of *Lactobacillus* as a clinical biomarker [44]. In a similar way, Gerede et al. (2024) [45] in their review discussed how the increase in relative abundance of *Lactobacillus crispatus* is associated with a greater protective effect in pregnant women and how other non-*Lactobacillus* bacterial species such as *Gardnerella* and *Prevotella* are associated with greater maternal morbidity. Therefore, our results are in agreement with recent literature.

Some recent studies have shown that the presence of bacteria favorable to the female microbiota were associated with a significant reduction in preterm births and unfavorable bacteria with increased preterm birth. Moreover, recent reviews on the subject of the vaginal microbiome indicate that women with a healthy microbiome, with many *Lactobacillus* spp., have fewer sexually transmitted infections, implantation failures, and preterm births, although these outcomes are also influenced by factors such as lifestyle, diet, genetics, obesity, smoking habits, race, and age, among others. Some gynecological diseases such as endometriosis and endometritis are also related to the imbalance of the microbiome and are unfavorable to pregnancy [27,28,29,30,31,32,33,34,35,36].

Recent studies [10] have investigated the role of the vaginal and endometrial microbiomes in endometrial receptivity and reproductive health. Although there is no consensus on whether a unique genital microbiome exists or whether the microbiome differs between the vagina and endometrium, evidence indicates that dominance of *Lactobacillus* spp. in the female reproductive tract is generally associated with eubiosis and higher chances of successful implantation and pregnancy. In contrast, bacterial vaginosis disrupts the vaginal microbiota and unbalances the immune system by activating vaginal dendritic cells. Both vaginal and endometrial dysbiosis can elevate pro-inflammatory cytokines, causing local inflammation and overactivation of the immune response. This can impair endometrial receptivity, reduce the likelihood of successful embryo implantation, or increase the risk of miscarriage.

Reviews emphasize the need for further studies on the vaginal and endometrial microbiome and fertility, especially in respect of categorizing which bacteria are dominant and which exert a positive influence on reproductive outcomes and which a negative influence [46,47].

The vaginal microbiota accounts for 9% of the total human microbiota and plays a central role in maintaining vaginal homeostasis in women (Golob et al., 2024) [48].

A lack of *Lactobacillus* and a high diversity of bacteria are associated with dysbiosis, which, together with immune dysregulation, may contribute to unexplained recurrent miscarriage and implantation failure.

Future research on vaginal dysbiosis should consider infertility alongside demographic factors such as ethnicity, obesity, lifestyle, and population location, as all influence the vaginal microbiome [49]. 

Male genital infections and in general the semen microbiome may also have an impact on the vaginal microbiome. The male and female microbiomes have interactions due to intercourse and, as shown by Mandar et al. [50] who compared the seminal and vaginal microbiomes of couples, numerous shared DNA sequences or phylotypes were found (85%).

In an older study by Borovkova et al. [51] vaginal samples prior to and after intercourse were analyzed and the presence of *Ureaplasma* spp. after sexual intercourse with males suffering from subclinical prostatitis was confirmed. Exchanges between the two microbiomes are undebatable. However, the mechanisms beyond the interactions of the microbiomes of the couple are still unknown, and whether the combination of the two microbiomes temporarily after intercourse could influence embryo implantation should be clarified by further research. Seminal fluid may temporarily alter the vaginal environment toward alkalinity, favoring pathogen proliferation and negatively affecting conception.

Non-invasive diagnostic methods and innovative treatments are under development. The Nugent score remains widely used for bacterial vaginosis diagnosis, but it is subject to examiner variability, emphasizing the need for molecular techniques. Therapeutic approaches under investigation include antibiotics, *Lactobacillus* supplementation, and vaginal microbial transplantation. Our review suggests that high relative abundance of *L. crispatus* may improve pregnancy outcomes, and metagenomic approaches such as whole shotgun sequencing could enhance resolution for identifying bacteria at genus and strain levels, as well as their functional capabilities.

## 5. Strengths and Limitations

One of the main strengths of this review is the number of studies evaluated in the meta-analyses, with a total of more than 900 women participating. The results in relation to reproduction were significant, with clinical pregnancy rates and live birth rates being higher in women with a favorable microbiome. Another strength is that the review only analyzed studies of the vaginal microbiome which used molecular methods. However, the study also has some limitations that should be mentioned. First, the quality of the studies identified was not high or had a high risk of bias. Second, not all the studies contained data on live birth rates, which limited our analysis. Third, in respect of our analysis of which species were associated with a successful pregnancy, raw data was only available in one study. Finally, A key limitation of our study is the heterogeneity of the included populations and methodologies, including differences in study design, diagnostic methods, degrees of vaginal dysbiosis, and racial/ethnic composition, which may affect the comparability of results

## 6. Conclusions

Women with more favorable microbiomes showed better reproductive outcomes with a higher clinical pregnancy rate, a higher live birth rate, and a lower rate of pregnancy loss although there was a low-quality bias. The high relative abundance, especially of *Lactobacillus crispatus*, may increase the success of pregnancy.

## Figures and Tables

**Table 1:** Characteristics of selected studies.

Author and Year (Alphabetic Order)	Country	Age Year (sd)	BMI Index k/mg^2^ (sd)	AMH	Abundance of *Lactobacillus* ssp.	Causes of Infertility	Detection Method	Main Inclusion Criteria	Conclusion
Eskew et al., 2021 [27]	USA	32.27 (3.8)	30.94 (8.1)	2.16 (1.15)	The importance of timing in the assessment of vaginal microbiome to determine its associations with reproductive outcomes	Male factor (33.3%) Unexplained (6.7%) Tubal factor (13.3%) Endometriosis (6.7%) Ovulatory dysfunction (6.7%) PCOS (26.7%) PAIN (6.7%)	16S rRN bacterial community structure is not predictive for embryo transfer	Molecular feature of microbiome analysis	Bacterial community structure is not predictive for embryo transfer
Karaer et al., 2021 [28]	Turkey	23–29	20.0–29.9	No data in the article	The abundance of Lactobacillus was lower in women who failed to become pregnant	Male factor (37.03%) Unexplained (45.37%) Others such as poor ovarian reserve and tubal factor (17.60%)	16S rRNA	Molecular feature of microbiome analysis	The high abundance of the genus Streptococcus was greater in the non-pregnancy group
Koedooder et al., 2019 [29]	The Netherlands	The majority of participants were from 30–35 years old	Most participants had a BMI from 20–29.9	No data in the article	There was a low abundance of Lactobacillus in women who failed to become pregnant and a high abundance of Lactobacillus in pregnant women	Male factor (71.6%) Other factors (17%)	16S rRNA	Molecular feature of microbiome analysis	Without a favorable microbiome, the implantation and subsequent embryo development seem to be compromised
Koort et al., 2023 [32]	Estonia	In the ART group, the average age of women was 34.1 and in the control group, it was 32.2	In the ART group, 75% of participants had a BMI below 25 (k/mg^2^) and 25% had a BMI above 25 (k/mg^2^)	No data in the article	Bacterial vaginosis community and with *L. iners*-predominant and *L. gasseri*-predominant microbiome had a lower ART success rate than women with *L. crispatus*-predominant microbiome	No data in the article	16S rRNA and Nugent score	Molecular feature of microbiome analysis	Disturbed microbiome in the reproductive tract in both partners may be one of the reasons for ART failure
Miyagi et al., 2023 [30]	Japan	35.6 (34.1)	22.7 (22.5)	3.53 (0.61)	Pregnant women present significantly higher proportions of *Lactobacillus* spp.	Fallopian tube factor (47.6%) Male factor (23.8%) Endometriosis (33.3%)	16S rRNA	Molecular feature of microbiome analysis	The balance between *Lactobacillus* and pathological bacterial abundance was associated with pregnancy from ART
Vainamo et al., 2023 [24]	Finland	32.9	24.9	2.6 (1.9)	Role of *L. crispatus* in the success of IVF-ET	Endometriosis (33.3%) Male factor (6.7%) Tubal factor (16.7%) Anovulation (13.3%) Unexplained (30%)	16S rRNA	Molecular feature of microbiome analysis	Most women who achieved pregnancy had an indication of holding a reservoir of this beneficial Lactobacillus in their reproductive tract
van den Tweel et al., 2024 [33]	The Netherlands	34	27.3	-	There was a tendency of more miscarriages based on positive BV status or community state type groups III and IV	Male factor (34%) Tubal factor (5%) Hormonal (8.5%) Endometriosis (15%) Unknown (34%)	16S rRNA	Molecular feature of microbiome analysis	Bacterial vaginosis does not significantly impact ongoing pregnancy rates but could affect miscarriage rates
Vergaro et al., 2019 [31]	Spain	41.5	24.4	24.4	Higher proportion of samples dominated by *L. crispatus* in women achieving live birth	No data in article	16S rRNA	Molecular feature of microbiome analysis	Vaginal microbiota profile on the day of the embryo transfer is not related to live birth rate in women receiving oocytes

**Table 2:** MaAsLin2 analysis of the taxa represented in Väinämö et al. (2023) [24] cohort and association with a successful pregnancy.

Taxon	Coefficient	*p* Value
*Lactobacillus crispatus*	1.802	0.003
*Streptococcus anginosus*	−0.708	0.060
*Lactobacillus* sp.	0.402	0.605
*Limosilactobacillus* sp.	−0.135	0.542
*Lactobacillus iners*	0.425	0.656
*Lactobacillus jensenii*	−0.269	0.705
*Fannyhessea vaginae*	−0.320	0.374
*Anaerococcus* sp.	0	0.999
*Dialister* sp.	0	0.999
*Gardnerella* sp.	0	0.999
*Enterococcus* sp.	0	0.999
*Anaerococcus prevotii*	0	0.999
*Anaerococcus tetradius*	0	0.999
*Dialister succinatiphilus*	0	0.999
*Enterococcus faecalis*	0	0.999
*Escherichia coli*	0	0.999
*Finegoldia magna*	0	0.999
*Gardnerella swidsinskii*	0	0.999
*Gardnerella vaginalis*	0	0.999
*Lactobacillus gasseri*	0	0.999
*Limosilactobacillus coleohominis*	0	0.999
*Megasphaera lornae*	0	0.999
*Parvimonas micra*	0	0.999
*Peptostreptococcus anaerobius*	0	0.999
*Peptostreptococcus stomatis*	0	0.999
*Porphyromonas asaccharolytica*	0	0.999
*Porphyromonas uenonis*	0	0.999
*Prevotella amnii*	0	0.999
*Prevotella bivia*	0	0.999
*Streptococcus agalactiae*	0	0.999
*Streptococcus anginosus*	0	0.999
*Streptococcus dysgalactiae*	0	0.999
*Streptococcus hominis*	0	0.999
*Veillonella atypica*	0	0.999
*Veillonella dispar*	0	0.999
*Veillonella ratti*	0	0.999

## Abbreviations

The following abbreviations are used in this manuscript:

IVF	In vitro fertilization
ART	Assisted reproductive technology
NGS	Next-generation sequencing
STI	Sexually transmitted infection
CTS	Community state type
